# Methodological Challenges of Digital PCR Detection of the Histone *H3* K27M Somatic Variant in Cerebrospinal Fluid

**DOI:** 10.3389/pore.2022.1610024

**Published:** 2022-04-12

**Authors:** Margarita Zaytseva, Natalia Usman, Ekaterina Salnikova, Agunda Sanakoeva, Andge Valiakhmetova, Almira Chervova, Ludmila Papusha, Galina Novichkova, Alexander Druy

**Affiliations:** ^1^ Dmitry Rogachev National Medical Research Center of Pediatric Hematology, Oncology and Immunology, Moscow, Russia; ^2^ Epigenomics, Proliferation, and the Identity of Cells, Department of Developmental and Stem Cell Biology, Institut Pasteur, Paris, France; ^3^ Research Institute of Medical Cell Technologies, Yekaterinburg, Russia

**Keywords:** cerebrospinal fluid, liquid biopsy, dPCR, cell-free DNA, CNS tumor, diffuse midline glioma, *H3* K27M

## Abstract

Cell-free DNA (cfDNA) in body fluids is invaluable for cancer diagnostics. Despite the impressive potential of liquid biopsies for the diagnostics of central nervous system (CNS) tumors, a number of challenges prevent introducing this approach into routine laboratory practice. In this study, we adopt a protocol for sensitive detection of the *H3* K27M somatic variant in cerebrospinal fluid (CSF) by using digital polymerase chain reaction (dPCR). Optimization of the protocol was carried out stepwise, including preamplification of the *H3* target region and adjustment of dPCR conditions. The optimized protocol allowed detection of the mutant allele starting from DNA quantities as low as 9 picograms. Analytical specificity was tested using a representative group of tumor tissue samples with known *H3* K27M status, and no false-positive cases were detected. The protocol was applied to a series of CSF samples collected from patients with CNS tumors (*n* = 18) using two alternative dPCR platforms, QX200 Droplet Digital PCR system (Bio-Rad) and QIAcuity Digital PCR System (Qiagen). In three out of four CSF specimens collected from patients with *H3* K27M-positive diffuse midline glioma, both platforms allowed detection of the mutant allele. The use of ventricular access for CSF collection appears preferential, as lumbar CSF samples may produce ambiguous results. All CSF samples collected from patients with *H3* wild-type tumors were qualified as *H3* K27M-negative. High agreement of the quantitative data obtained with the two platforms demonstrates universality of the approach.

## Introduction

High-grade gliomas are rare tumors which constitute 10%–15% of the central nervous system (CNS) neoplasms in pediatric patients [1]. Clinical characterization of midline gliomas with infiltrative growth is conventionally limited to a diagnosis based on neuroimaging data (including topography, relative signal intensity, and specific patterns of the contrast enhancement), whereas proper histological and molecular verification is complicated, given the tumor location [2]. However, in many cases, the analysis of clinical and radiological parameters cannot provide reliable grading of a CNS tumor.

Basic findings of the last decade have substantially advanced the understanding of molecular pathogenesis of glial tumors in pediatric patients. Wu et al. identified K27M^1^ substitution in histone 3.3 in 60% of diffuse intrinsic pontine gliomas [3]. Further studies of molecular landscapes of high-grade gliomas confirmed the high prevalence of *H3* K27M variant in diffuse midline gliomas (DMG). This variant is observed in up to 94% of diffuse brainstem gliomas, 65% of thalamic gliomas, and 60% of spinal cord gliomas [4]. Moreover, the presence of *H3* K27M mutation is a very unfavorable prognostic factor. Accordingly, a distinct nosological entity ‘Diffuse midline glioma *H3* K27M-mutant’ was added to the 2016 update of the WHO classification of CNS tumors [5]. Verification of such diagnoses implies determination of the *H3F3A* mutational status. Importantly, the detection of *H3* K27M mutation in an infiltrating midline glioma implicates assignment of the tumor as WHO grade IV by definition.

Molecular profiling of malignant gliomas, enabled by modern advances in molecular oncology, allows ultimate specification of the diagnosis and selection of optimal therapies targeted at specific aberrations. Molecular inclusion criteria are increasingly used in clinical trials. The *H3* K27M substitution, in particular, is a prerequisite for the inclusion in currently running trials of anti-DRD2 agent ONC201 as a therapeutic option for *H3* K27M-mutant DMG (NCT03295396 and NCT02525692, both enrolling adult patients, and NCT03416530 enrolling pediatric patients). In this regard, the possibility of using liquid biopsy—an approach based on the presence of cell-free DNA (cfDNA) of tumor cells in biological fluids—is of particular interest. Repeated sampling of body fluids (plasma, serum, cerebrospinal fluid, etc.) provides access to the dynamics of genetic aberrations arising at different stages of tumorigenesis and allows monitoring of the therapy effectiveness [6].

Plasma is certainly the most common source of cfDNA for analysis. Representation of somatic mutations in plasma has been demonstrated for various solid tumors [7]. In 2016, determination of *EGFR* mutations in plasma specimens was approved by FDA as a companion diagnostic test in non-small cell lung cancer patients with poorly accessible tumors [8]. Liquid biopsies can be highly informative for molecular profiling. For instance, osimertinib recipients with *EGFR* mutations determined by using either liquid or conventional biopsy showed similar survival rates [9]. In 2019, FDA approved a diagnostic protocol involving determination of *PIK3CA* variants in cfDNA isolated from plasma of the patients with ER-positive HER2-negative locally advanced or metastatic breast cancer [10]. For patients with CNS tumors, penetration of tumor cfDNA into the blood circulation is hampered by the blood-brain barrier, which excludes the use of plasma for detection of somatic variants in CNS [11,12].

Examination of cerebrospinal fluid (CSF) samples is a promising diagnostic tool for neurooncology. However, the search for tumorigenic genetic aberrations in CSF is nontrivial due to the low concentrations of cfDNA [13,14]. The applicability is further limited by analytical sensitivity of the methods used for the analysis of somatic mutations in cfDNA. Mutations with extremely low allele burden are best identified with digital PCR (dPCR). Currently available dPCR platforms involve droplet- (RainDance Technologies and Bio-Rad), sealed chip- (Thermo Fisher), microfluidic chamber- (Fluidigm), or nanoplate-based workflows (Qiagen). To perform the experiments, we opted for one of the most common Droplet Digital™ PCR platform (Bio-Rad) and a new QIAcuity Digital PCR System (Qiagen).

The aim of this study was to develop a reliable cross-platform dPCR protocol for detection of *H3* K27M variant in cfDNA isolated from CSF of pediatric patients with CNS tumors.

## Patients and Methods

### Study Design

**FIGURE 1:**
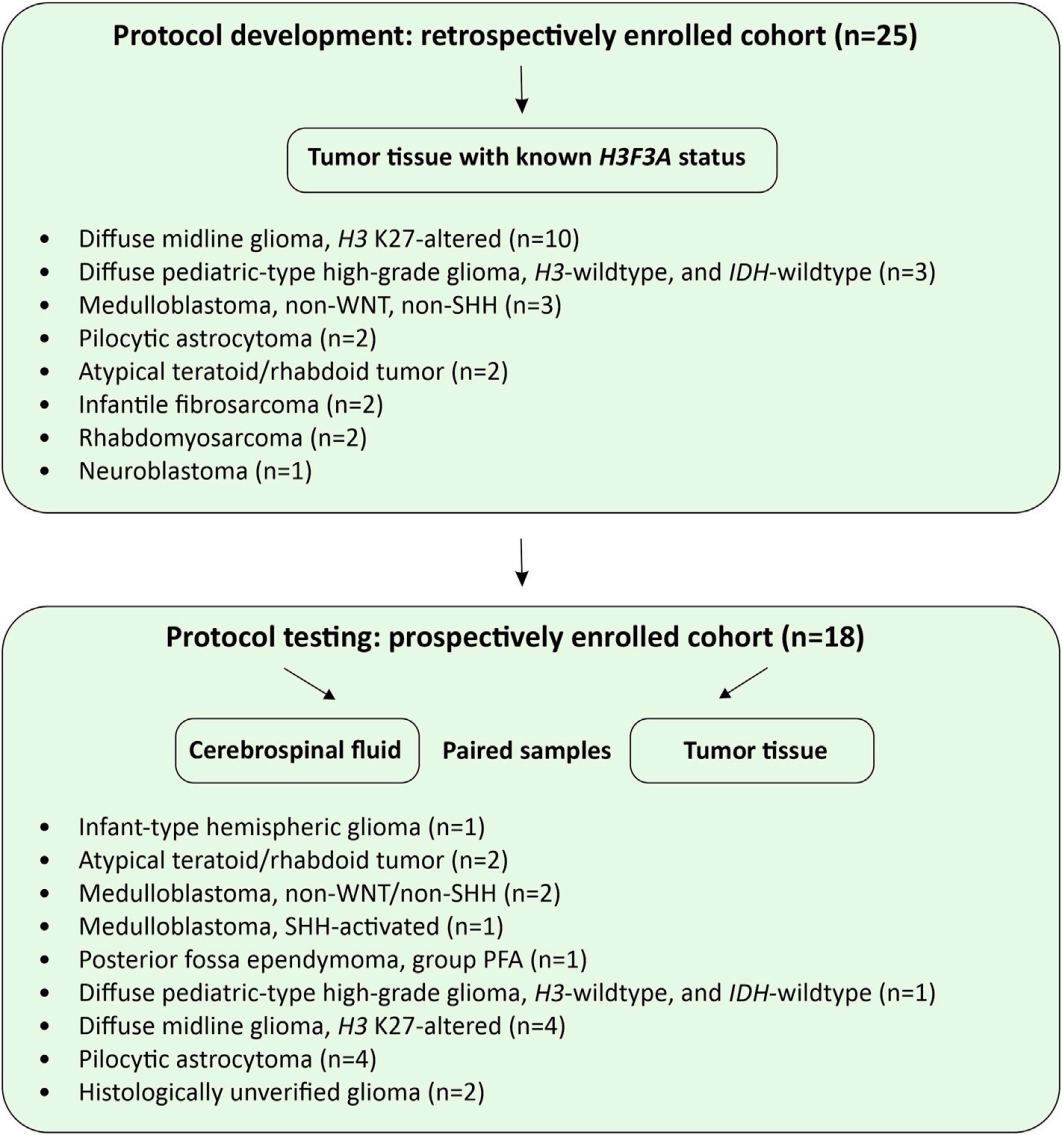
Study design. The flow charts illustrate protocol development and pilot testing on CSF-derived cfDNA.

Overall design of the study is presented in Figure 1. A retrospective cohort (*n* = 25) was intended for the protocol development and its analytical characterization on formalin fixed/paraffin embedded (FFPE) tumor tissues. For a prospective cohort (*n* = 18), FFPE and CSF samples were matched for diagnostic characterization and clinical validation of the protocol. All patients of the prospective cohort, presenting with newly diagnosed or recurrent radiographically-confirmed CNS tumors, were enrolled in the Molecular Diagnostics in Pediatric Neurooncology and Liquid Biopsies Project at the Dmitry Rogachev National Medical Research Center of Pediatric Hematology, Oncology and Immunology. An exclusion criterion was complete surgical resection (R0) determined on the basis of postoperative MRI data, unless CSF sampling has occurred during or before surgery. The Informed Consent for participation in the study was obtained from parents/guardians of all patients. The study was approved by the Institutional Review Board at the Dmitry Rogachev Center.

### CSF Collection and Characterization

Clinical and analytical data for CSF donors (prospective cohort) are summarized in Table 1. CSF samples (1.0–13.3 ml, *n* = 19), collected either intraoperatively, or through a ventricular access device, or by lumbar puncture, were clarified by centrifugation at 1,100 g for 10 min and stored in 1 ml aliquots at −80°C. The samples were thawed immediately before cfDNA isolation. Cell-free DNA was isolated with the use of QIAmp Circulating Nucleic Acid Kit (Qiagen, the Netherlands). Concentrations of amplifiable fragments were measured by DNA Fragmentation Quantification Assay (EntroGen, United States). The cfDNA profiles were analyzed with the use of Cell-free DNA ScreenTape Assay Reagent Kit (Agilent Technologies).

**TABLE 1:** Patient data^a^ with specification of tumors and cfDNA content in CSF.

#	Age of onset	Gender	Integrated diagnosis^b^	Tumor volume, cm^3^ ^c^	Time of CSF collection	CSF collection procedure	CSF sample volume, ml	37 bp fragment concentration, copies/µl	150 bp fragment concentration, copies/µl	300 bp fragment concentration, copies/µl	*H3* K27M in tumor tissue	*H3* K27M in CSF
1	5 months	Female	Infant-type hemispheric glioma, *ETV6-NTRK3*-positive	345	Primary	VAD	3	16,793	15,170	8,583	-	-
2	3 months	Female	Atypical teratoid/rhabdoid tumor, *SMARCB1*-mutant	99.5	During therapy	VAD	2.4	2,972	1,486	546	-	-
3	2 years 10 months	Male	Atypical teratoid/rhabdoid tumor, NOS	9.5	During therapy	VAD	2.8	676	417	306	-	-
4	1 year 11 months	Female	Anaplastic medulloblastoma, non-WNT/non-SHH	12.2	During therapy	VAD	4	361	122	101	-	-
5	5 years 5 months	Male	Desmoplastic medulloblastoma, SHH-activated, *TP53*-wildtype, *PTCH1*-mutant	0.44	Metastatic relapse	VAD	2.5	53	35	20	-	-
6	11 years 5 months	Male	Medulloblastoma, non-WNT/non-SHH	26.3	Primary	IO	2	0	0	0	-	NA
7	5 years 2 months	Male	Posterior fossa ependymoma, group PFA	2.2	Local relapse	IO	4	973	683	669	-	-
8	10 years 8 months	Male	Diffuse pediatric-type high-grade glioma, *H3*-wildtype, and *IDH*-wildtype	140	Primary	IO	4	64	47	142	-	-
9	11 years 11 months	Female	Diffuse midline glioma, *H3* K27-altered	37.4	After surgery	LP	3	16	10	0	*+*	*+*
10	5 years 1 month	Male	Diffuse midline glioma, *H3* K27-altered	16	Primary	LP	2	1	3	4	+	?
11	5 years 7 months	Male	Diffuse midline glioma, *H3* K27-altered	14	Primary	IO	13.3	66	108	87	+	+
								320	165	137		
						LP	1	4	7	3		?
12	11 years 2 months	Male	Diffuse midline glioma, *H3* K27-altered	87	Primary	IO	10	27	14	6	+	+
								19	8	9		
13	6 years 9 months	Male	Optic chiasm pilocytic astrocytoma, NOS	10	Primary	VAD	5	106	81	51	NA	-
14	1 month	Male	Pilocytic astrocytoma, *KIAA1549* (exon 15)—*BRAF* (exon 9)—positive	134	During therapy	VAD	4.8	387	108	67	-	-
15	7 years 3 months	Male	Pilocytic astrocytoma, *KIAA1549* (exon 16)—*BRAF* (exon 9)—positive	2.9	Primary	LP	3.5	0	0	0	—	NA
16	9 months	Female	Pilocytic astrocytoma, *KIAA1549* (exon 15)—*BRAF* (exon 9)—positive	74	Local relapse	IO	1.2	0	0	0	—	NA
17	8 years 8 months	Female	Histologically unverified bilateral thalamic tumor with midbrain extension (suspected high-grade glioma)	142	Primary	VAD	2	9	6	0	NA	-
18	4 years 10 months	Female	Histologically unverified tumor of the right cerebral peduncle and basal ganglia on the right (suspected low-grade glioma)	16.4	Primary	LP	2	2	0	0	NA	NA

a Sorted roughly by morphological assignment.

b Based on the 2021 WHO Classification of Tumors of the Central Nervous System [15].

c Tumor volume at the time of CSF collection.

NA, no data available; VAD, ventricular access device; IO, intraoperatively; LP, lumbar puncture.

### Protocol Development

The protocol was adapted and optimized using the Droplet Digital™ PCR platform (Bio-Rad) for FFPE tumor tissues specimens of the retrospective cohort (*n* = 25, Figure 1) with the known *H3F3A* K27M status determined by Sanger sequencing (Supplementary Table S1). Genomic DNA was isolated from the tumor tissues using FFPE RNA/DNA Purification Plus Kit (Norgen Biotek, Canada). The degree of fragmentation corresponding to cfDNA was modeled by shearing in a Covaris M220 Focused-Ultrasonicator (Covaris, United States) in a standard 150 bp mode. The DNA was subsequently purified and concentrated with AMPure XP beads (Beckman Coulter, United States) and quantified on a Qubit 3.0 fluorometer (Thermo Fisher Scientific, United States) with Qubit dsDNA BR Assay Kit (Thermo Fisher Scientific). Representation of different fragment lengths (37 bp, 150 and 300 bp) was evaluated using DNA Fragmentation Quantification Assay (EntroGen, United States) and additionally by automated electrophoresis in a TapeStation 2200 system (Agilent Technologies, United States) with the use of High Sensitivity DNA ScreenTape Analysis Reagent Kit (Agilent Technologies).

Due to the expected low concentrations of the *H3F3A* target in cfDNA, the preamplification step was introduced to increase the sensitivity of dPCR assay. The reactions were set in 25 µl of 1× PCR buffer B (Syntol LLC, Russia) containing 50 nM of primers, 0.25 mM dNTPs, 2.5 mM MgCl_2_ and 0.1 U/µl Taq polymerase (Syntol LLC) with 50–100 copies of 150 bp DNA fragments per reaction. Preamplification was run in a T100 thermal cycler (Bio-Rad) at 98°C for 1 min, followed by eight cycles of 98°C for 10 s, 58°C for 30 s and 72°C for 30 s, followed by 72°C for 2 min. The product was diluted 1:1 with TE buffer and used in dPCR with LNA-modified allele-specific probes. The reactions were set in duplicates, each comprising 20 µl of 1× ddPCR Supermix for Probes (No dUTP) (Bio-Rad, United States) with the primers and probes added to final concentrations of 1,800 nM and 500 nM, respectively. The process was run in a C1000 Touch Thermal Cycler (Bio-Rad) equipped with a QX200 Droplet Digital PCR (ddPCR) system (Bio-Rad) at 95°C for 10 min, followed by 40 cycles of 94°С for 30 s (ramp 2°C/s) and 57°C for 60 s (ramp 2 °C/s), followed by 98°C for 10 min. The data were processed and analyzed in QuantaSoft Version 1.6.6.0320 (Bio-Rad). Fluorescence thresholds (respectively, 2,000 and 1,500 RFU for FAM and HEX) were set manually.

Oligonucleotide sequences of primers and probes previously published in [16] were as follows: forward *H3* K27M: 5′-GGT​AAA​GCA​CCC​AGG​AAG-3′; reverse *H3* K27M: 5′-CAA​GAG​AGA​CTT​TGT​CCC-3′; wild-type probe: 5′-HEX-TC^+^GC^+^A^+^A^+^GA^+^GT^+^GC-RTQ1-3′; K27M probe: 5′-6-FAM-TC^+^GC^+^A^+^T^+^GA^+^GTGC-RTQ1-3′, with “locked” nucleic acid-modified (LNA) bases indicated by^+^. LNA-modified allele-specific probes are known to increase dPCR specificity. Distinctive structural feature of LNA residues is the methylene bridge between atomic positions O2′ and C4′ of furanose, which locks the sugar in 3′-*endo* conformation thus enhancing hybridization of the probe with the template. Introducing LNA-monomers into oligonucleotide sequences significantly improves their discriminating capacity in the recognition of single-nucleotide substitutions [17].

### Compatibility of the Developed Protocol With QIAcuity™ Digital PCR System

The optimized protocol was tested for compatibility with the nanoplate-based digital PCR system (Qiagen) technologically different from the water-oil emulsion droplet-based PCR platform (Bio-Rad). The mixtures were set up in duplicates with QIAcuity Probe PCR Kit in Nanoplate 8.5K 96-well plates (Qiagen). The reactions, containing 3 µl 4× Probe PCR Master Mix, 1.2 µl primer–probe mix, 5 µl DNA template, and 2.8 µl RNase-free water in 12 µl total volume each, were run in a QIAcuity One instrument (Qiagen). The analysis was carried out with the use of QIAcuity Software Suite (Qiagen). Fluorescence thresholds for optimal resolution and cloud clustering, selected manually to fit all reactions, constituted 40 and 20 RFU for FAM and HEX, respectively. To enhance the accuracy of concentration measurements, the Volume Precision Factor (VPF), adjusting for tiny variations in nanoplate geometry, was applied as recommended by the manufacturer.

### Statistics

To quantitatively describe the dependence of dPCR outputs on reaction parameters (preamplification cycle number and template copy number), regression lines and determination coefficients were calculated in Microsoft Excel 2016. Bland–Altman test was used to assess quantitative agreement between the Droplet Digital™ PCR (Bio-Rad) and the QIAcuity™ Digital PCR System (Qiagen) based on the mean difference and standard deviation. The estimated concentrations of the target in starting samples were expressed in log_10_ copies/µl. Non-paired measurements (i.e., with missing value for an alternative platform) were excluded from the analysis.

## Results

### Optimization of the Protocol

The *H3F3A* target preamplification protocol, as well as ddPCR settings and conditions, were optimized in order to increase the analytical sensitivity and specificity of the *H3* K27M variant determination. The optimality was defined as a combination of high number of positive droplets with robust cloud separation and minimal “rain” effects (a fraction of droplets showing sub-threshold fluorescence).

**FIGURE 2:**
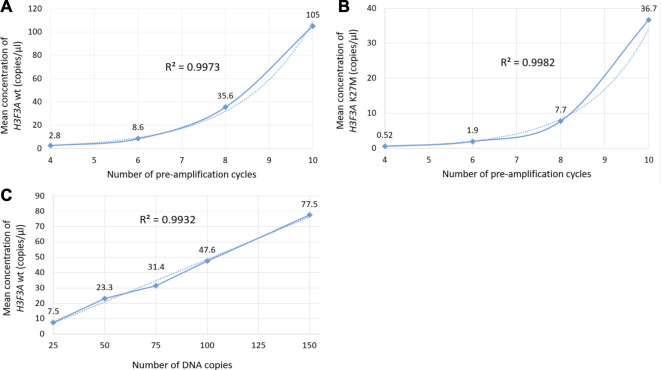
Evaluation of ddPCR outputs for different reaction conditions. Plots **(A,B)** represent the dependence of the outputs of the *H3F3A* K27M mutant and *H3F3A* wild-type targets (for the samples with and without mutation, respectively) on the preamplification cycle number with 50 copies of DNA target at the start of the reaction. Plot **(C)** represents the dependence of the yields of wild-type target on the number of DNA copies at the start of preamplification (eight cycles). The dotted lines are estimated trends, *R* ^2^ are correlation coefficients.

**FIGURE 3:**
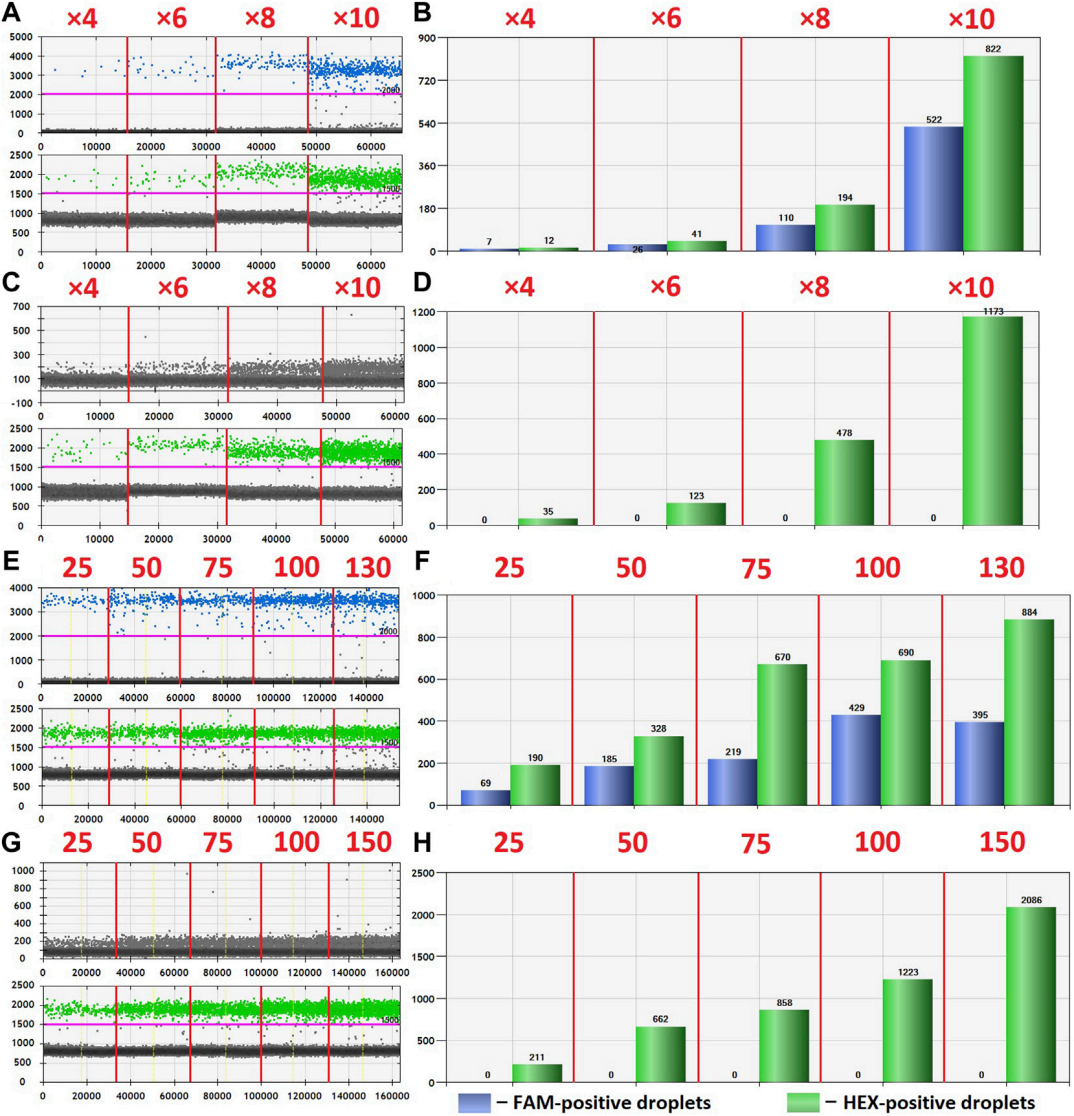
Primary ddPCR data for different reaction conditions, visualized in QuantaSoft Version 1.6.6.0320 (Bio-Rad). Varying the preamplification cycle number with 50 copies of the target fragment at the start of the reaction: **(A,B)**—sample with *H3* K27M mutation; **(C,D)**—sample without *H3* K27M mutation (wild-type *H3F3A* allele); preamplification cycle numbers (4, 6, 8, or 10) are indicated at the top of the panels. Varying the number of target DNA copies at the start of the reaction with eight cycles of preamplification: **(E,F)**—sample with *H3* K27M mutation; copy numbers of the target at the start of the reaction (25, 50, 75, 100, or 130) are indicated at the top of the panel; **(G,H)**—sample without *H3* K27M mutation (wild-type *H3F3A* allele); copy numbers of the target at the start of the reaction (25, 50, 75, 100, or 150) are indicated at the top of the panel.

The optimization was carried out by varying preamplification cycle number (4, 6, 8, or 10), annealing temperatures and concentrations of the oligos, as well as the number of DNA copies at the start of the reaction. The exponential dependence of the ddPCR-detectable amount of DNA target on the preamplification cycle number (Figures 2A,B) allows significant boost in representation of the target. Figure 2C demonstrates the dependence of ddPCR outcomes on the number of DNA template copies at the start of the reaction when using eight cycles of preamplification of the *H3F3A* fragment. The analyses were set in duplicates for each sample. The data indicate that eight cycles of preamplification of the *H3F3A* gene fragment are necessary and sufficient for optimal cluster density and accurate interpretation of the results of ddPCR with 50–100 copies of the template at the start of preamplification. Representative examples of primary ddPCR data for different reaction conditions are shown in Figure 3.

The protocol was further optimized by adjusting annealing temperatures of ddPCR primers (within the range of 53–61°C) and final concentrations of the primers and probes in the reactions. Oligonucleotide concentrations were tested extensively within the range of 900–2,700 nM with a step of 300 nM for the primers and 250–750 nM with a step of 125 nM for the probes. Reducing the probe concentration to values below 500 nM resulted in the reporter fluorescence in HEX channel too low for reliable cloud separation. Elevated concentrations of oligonucleotides in the mixture increased the overall intensity of fluorescence but negatively affected specificity of the reaction. High concentration of the FAM-labeled fluorescence probe specific for the H3 K27M allele led to the non-specific annealing of the probe to the normal allele. This resulted in the ambiguous appearance of the small amount (less than 50 in two technical replicates) of positive droplets with fluorescence above 2,000 RFU in the FAM channel in samples with absence of the *H3* K27M substitution. Such results were considered as false positive (ambiguous sample). A sample containing at least 50 droplets with above-the-threshold fluorescence in the FAM channel and reproducible in two technical replicates was considered positive. In contrast, a sample containing more than 50 droplets with above-the-threshold fluorescence in the HEX channel and less than 50 droplets with above-the-threshold fluorescence in the FAM channel in the total number of duplicates and reproducible in two technical replicates was considered negative.

**FIGURE 4:**
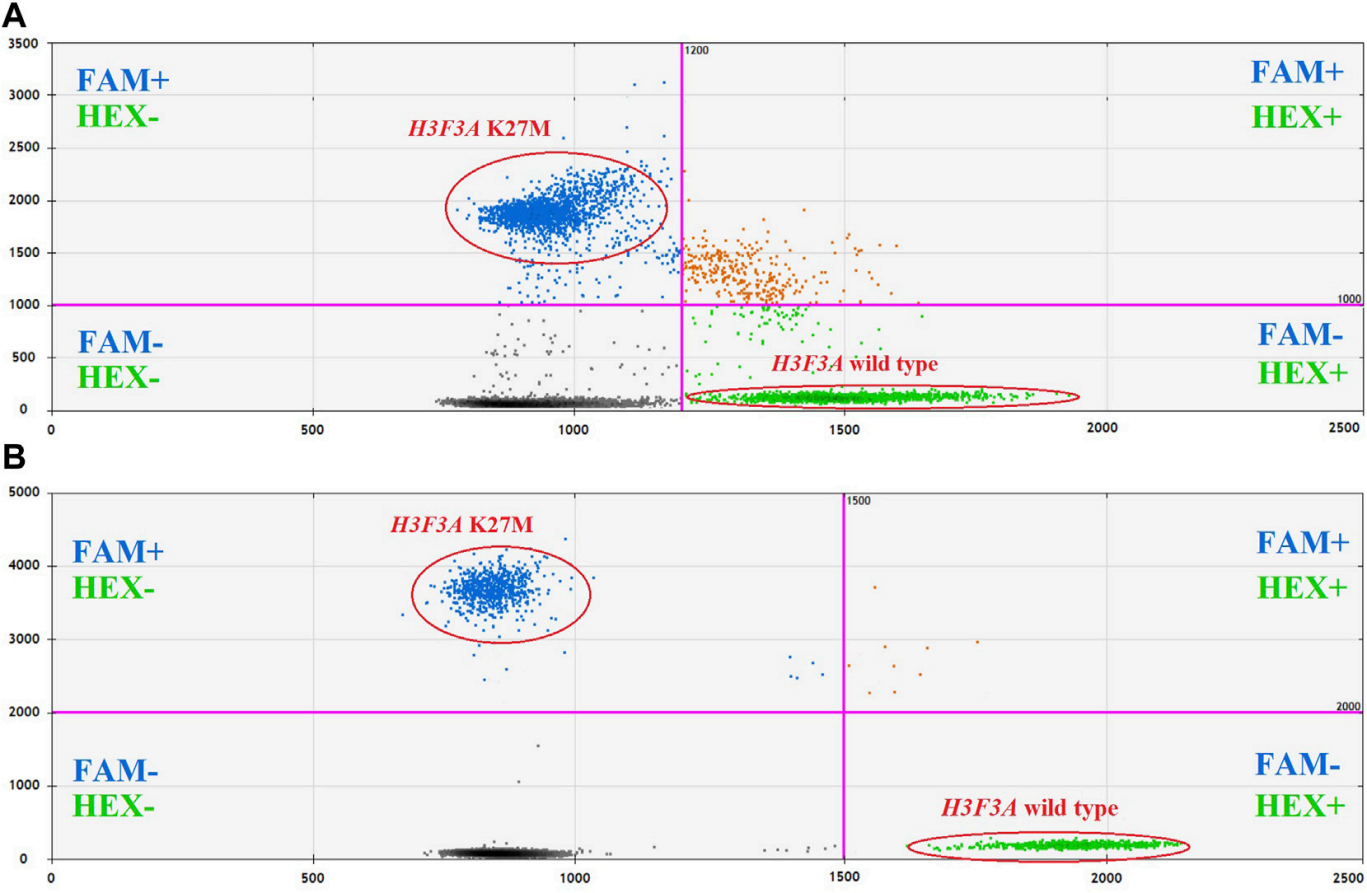
2D cluster plots of dual channel droplet fluorescence, Channel 1 (FAM) corresponding to *H3F3A* K27M vs. Channel 2 (HEX) corresponding to wild-type *H3F3A* (pt. 11, tumor DNA). Typical data obtained before **(A)** and after **(B)** optimization of the protocol.

The optimal fluorescence levels and maximal ddPCR efficiency were achieved at eight cycles of preamplification with 50–100 copies of DNA template and annealing temperatures of 58°C and 57°C for preamplification and ddPCR, respectively. The clearest cluster separation efficiency was achieved with 1,800 nM of the primers and 500 nM of the probes. The uniform threshold values of the fluorescence intensity, above which the droplets were regarded as positive, were set manually. The thresholds for FAM and HEX signals constituted 2,000 and 1,500 RFU, respectively. Typical results of ddPCR obtained before and after the optimization experiments are shown in Figure 4. The optimized parameters of the developed ddPCR-based system are described in the Patients and Methods section.

### Analytical Characterization of the Protocol

**FIGURE 5:**
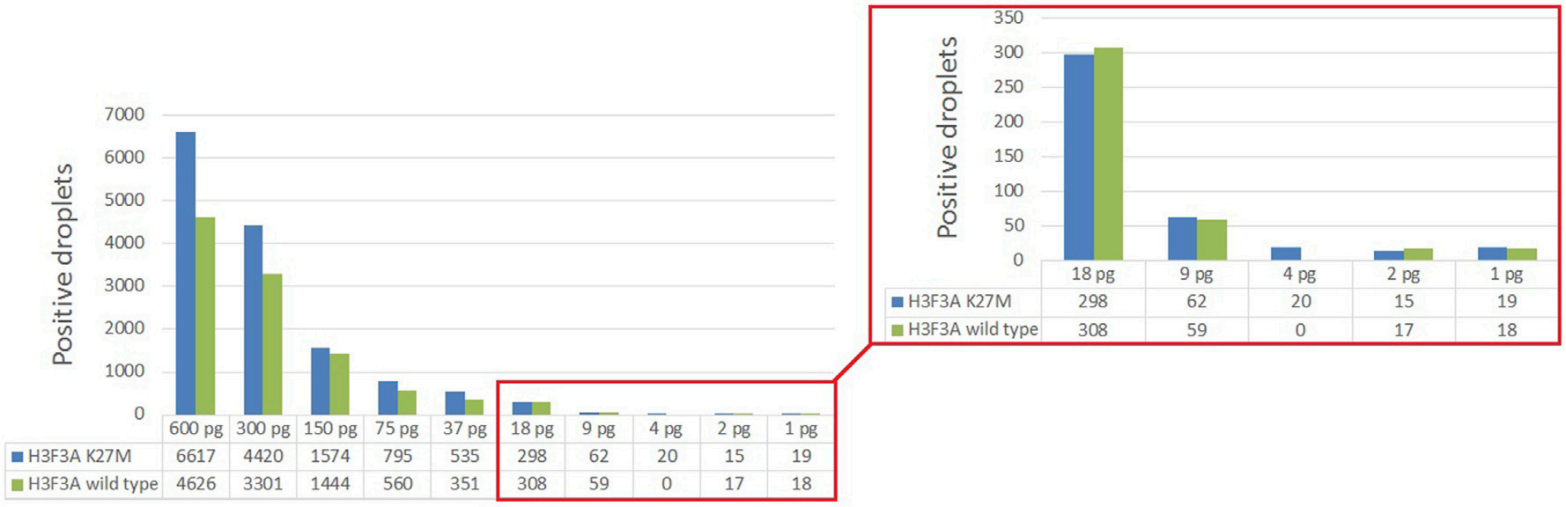
Determining the lower limit of *H3* K27M detection by ddPCR.

Analytical sensitivity of the modified ddPCR protocol was tested using a series of 10 sequential two-fold dilutions of genomic DNA comprising the studied *H3* K27M variant, with concentrations descending from 600 to 1 pg/μl of DNA at the start of preamplification. The reactions were carried out in two technical replicates. The sensitivity threshold was determined by minimal dilution affording at least 50 droplets with above-the-threshold fluorescence in at least one of the channels in total counts for the duplicates (Figure 5). The limit of detection for the *H3* K27M variant determined with the use of optimized ddPCR protocol was 9 pg of DNA (corresponding to 3 haploid genomes).

Analytical specificity of the developed ddPCR-based approach was tested using a selection of genomic DNA samples isolated from tumors with known *H3* K27M status determined by Sanger sequencing (the retrospective cohort, Figure 1). The lack of positive signals for *H3* K27M-negative samples confirmed full specificity of the developed protocol (see Supplementary Table S1).

### Primary Validation for the Diagnostics

PCR-based assay of representation/integrity of amplifiable cfDNA in the studied samples revealed wide variation. For pts. 6, 15, 16, and 18, insufficient analytical quality of the isolates was indicated by the absence of ≥150 bp DNA fragments; these samples were excluded from further analysis as non-informative. In other samples, the concentrations ranged from 3 to 15,170 copies/µl with a median of 81 copies/µl for the working fragment length of 150 bp (Table 1).

The developed ddPCR-based system was applied for determination of the *H3* K27M somatic variant in cfDNA isolated from CSF. The analysis of CSF-derived cfDNA was validated against matching samples of FFPE tumor tissue by Sanger sequencing. The studied variant was identified in specimens #9–12 implicating the diagnosis of DMG *H3* K27-altered. FFPE specimens #1–8 and #14–16 were *H3* K27M-negative, and no solid bioptates were available for pts. 13, 17, and 18 (Table 1).

**FIGURE 6:**
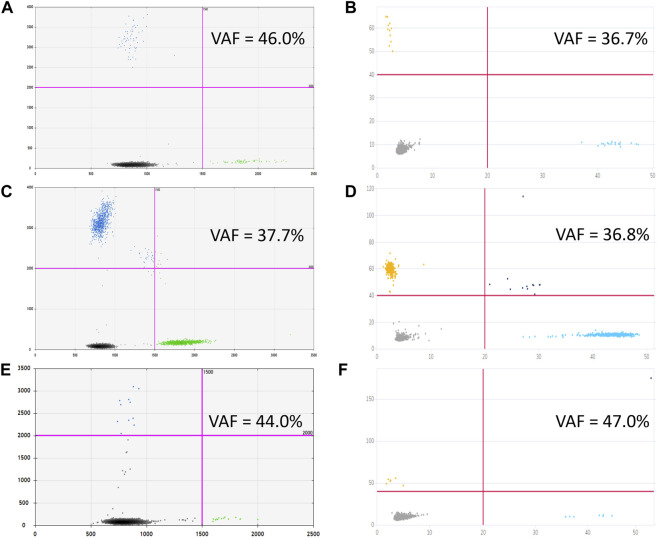
Graphical outcomes of dPCR with **(C,D)** and without **(A,B)** preamplification step, performed on Droplet Digital™ PCR **(A,C)** and QIAcuity™ Digital PCR **(B,D)** platforms for ventricular CSF, to be compared with cumulative plots, comprising four reaction replicas each, obtained from lumbar CSF with preamplification [Droplet Digital™ PCR, **(E)**, and QIAcuity™ Digital PCR, **(F)**].

In sum, 14 ventricular (6 collected intraoperatively and eight from a ventricular access device), and 5 lumbar CSF samples were analyzed, corresponding to 18 patients with CNS tumors grade I–IV. In three out of four *H3* K27M-positive cases, the mutation was unequivocally identified in ventricular (pts. 11 and 12) or lumbar CSF (pt. 9). For pt. 11, both ventricular and lumbar CSF samples were available. Interpretation of the results for ventricular CSF was straightforward whereas analysis of the lumbar sample produced ambiguous results—solitary high-intensity signals in two channels (i.e., for both alleles) independently of dPCR platform. Comparative representation of these data is given in Figure 6. It is important to note that variant allele frequencies (VAFs) measured by these two platforms were identical and did not depend on the use of preamplification step. At the same time, VAFs in CSF were considerably lower compared with tumor tissue (37% vs. 51.3%).

For pt. 10, only lumbar CSF with extremely low yields of cfDNA was available for the analysis, producing the same uninterpretable result despite preamplification.

All CSF samples from patients with *H3* K27M-negative tumors (*n* = 11) produced negative results in dPCR for *H3* K27M substitution, which indicates high diagnostic specificity of the developed protocol.

### Diagnostic Sensitivity Assay

**FIGURE 7:**
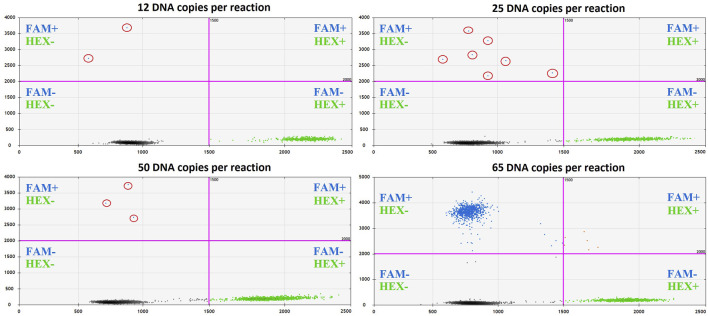
The impact of cfDNA template load on the ddPCR result (sample #9).

The accuracy of quantitative analysis for samples containing small amounts of genetic material (10–100 copies of DNA template) is limited by stochastic sampling. For small DNA copy numbers, the variation in the number of mutant alleles taken from the extracted volume of cfDNA and introduced into the preamplification reaction is described by the classical Bernoulli test scheme, and for copy numbers of 100 and above, according to the Moivre-Laplace limit theorem, by the Gaussian (normal) distribution [18]. In ordinary terms, with increasing concentrations of cfDNA, the probability of capturing the *H3* K27M mutant allele into the analyzed pool of molecules increases. To describe the influence of the initial abundance of the mutant allele on the ddPCR outcome, the *H3F3A* target was amplified from 65, 50, 25, or 12 copies of the template (sample #9) at the start of preamplification. Comparative representation of the outcomes for different amounts of the template is given in Figure 7.

The number of copies of the DNA fragment-of-interest produced in the reaction at time *t* can be calculated as follows:

n=f(k)k⁡exp(tv),

where *n* is DNA copy number after PCR, *k*—DNA copy number at the start of the reaction, *v*—reaction rate, *t*—time, and *f(k)* is a threshold function of *k*, *f(k)* = {0 at *k* < *h*; 1 at *k*≥*h*}, with *h* regarded as the threshold value.

The known number of DNA copies at the start of the reaction enables estimation of the expected number of amplified DNA fragments. Considering that *k* = 65 DNA copies at the start of the reaction corresponds to *n* = 1,574 positive droplets in the FAM channel, we get exp(*tv*) = 24.2. Accordingly, at *k* = 50, the expected number of amplified molecules will be *n* = 1,210, but only if *k* is not below the threshold value *h*; otherwise, *n* = 0. Thus, the empirical value of *n* = 3 is an artifact and should be replaced with a zero value. Similarly, at *k* = 25, the expected number of amplified copies will be *n* = 605, and at *k* = 12 it will be *n* = 290. In accordance with this model, the numbers of positive droplets (measured in the FAM channel corresponding to the mutant allele) are valid only for the case of 65 copies of the template at the start of the reaction. However, this value could vary depending on VAF in the tumor and should not be considered as a threshold level, but only as the explanation for the appearance of single positive droplets. It’s important to note that in the described case #9 *H3* K27M VAF was relatively low—14%.

The appearance of the low amount (<2) of the positive droplets has been seen after preamplification in several samples with confirmed H3 wild type status belonging to patients with the infant-type hemispheric glioma, *ETV6-NTRK3*-positive (sample #1), posterior fossa ependymoma, group A (sample #7), diffuse pediatric-type high-grade glioma, H3-wildtype, and IDH-wildtype (sample #8) and pilocytic astrocytoma, *KIAA1549* (exon 15)—*BRAF* (exon 9)—positive (sample #14). The presence of such droplets confirms their artefactual origin. The explanations of the appearance of the solitary droplets are either spontaneous cleavage of the fluorescence probe or its unspecific binding to the wild-type allele.

At the same time, CSF derived cfDNA samples from patients with *H3* K27M altered tumors provided adequate positive results beginning from significantly lower amounts of the input DNA (19 copies/µl, sample#11.1 and 31 copies/µL, sample#12.1) compared to 65 copies/µl. Remarkably, the VAF in both of this cases were higher (22 and 60%, respectively), comparing to the sample #9. Single mutant allele copies in the starting material could provide solitary positive droplets (samples#10 and 11.5), but extremely low overall cfDNA input prevents an unambiguous interpretation of the results.

### Comparative Evaluation of Different Technological Platforms

The optimized protocol was tested using an alternative digital PCR technology platform (QIAcuity™) to evaluate its universality. Analytical consistency between the platforms was evaluated using Bland–Altman method. The analysis was performed on cfDNA with and without preamlification step. A total of 43 paired measurements were analyzed; four measurements, performed on a single dPCR platform, were excluded from the analysis given the lack of pairness. Due to high volume of CSF collected from patients 11 and 12, nucleic acids were isolated in quadruplicates and in duplicates, respectively (all included in the analysis); see Supplementary Table S2 for the raw data.

**FIGURE 8:**
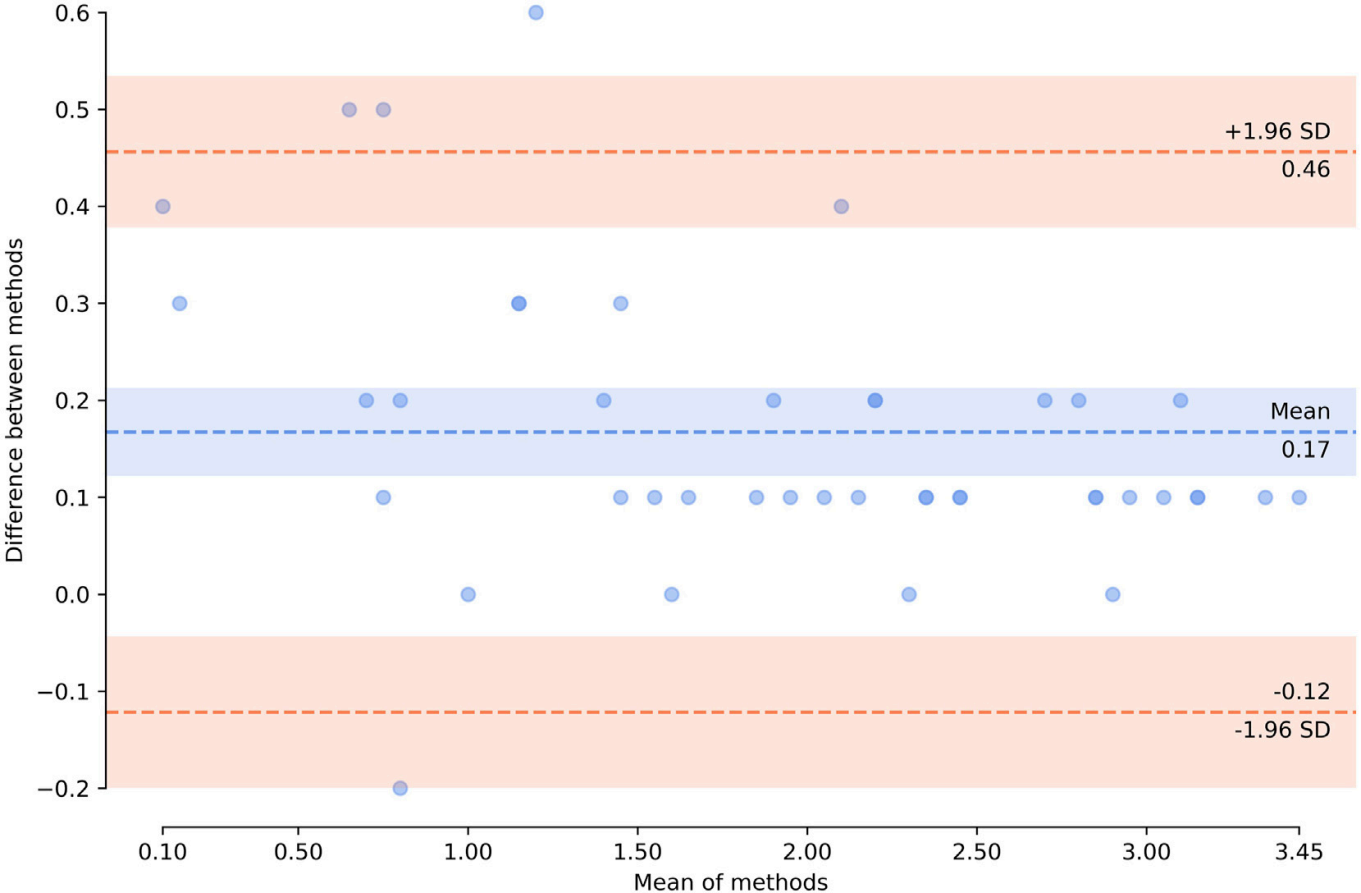
Bland–Altman plot showing agreement of the results obtained by using two technological platforms—Droplet Digital™ PCR and QIAcuity™ Digital PCR System. Horizontal axis represents log_10_ (mean concentration of the target, copies/µl, in the starting sample). Vertical axis shows log_10_ (difference between the methods); blue dashed line represents the mean difference; red dashed lines represent the limits of agreement (mean difference ±1.96 × SD). Zero concentration values were excluded from the analysis. The plot was created using pyCompare (http://doi.org/10.5281/zenodo.1238915).

Bland-Altman analysis (Figure 8) showed that the bias (mean difference) was 0.17 log_10_ copies/µl, with a standard deviation (SD) of 0.15 log_10_ copies/µl. For the vast majority of measurements (39 of 43, 90.7%), the results showed marked pairwise consistency of concentration values for mutant and wild-type alleles obtained with the two platforms, falling within the ±1.96 SD limit of agreement (−0.12…0.46 log_10_ copies/µl). The rest of the differences (4 of 43, 9.3%) were outside the limits of agreement. For three of these outliers (two wild-type and one comprising the mutation), concentration values obtained with QIAcuity exceeded the differences, ranging from 0.52 to 0.60 log_10_ copies/µl. For one of the outliers, the concentration of mutant allele obtained with QX200 was higher (−0.22 log_10_ copies/µl). It should be noted that all differences falling outside the limits of agreement corresponded to non-preamplified cfDNA. Concentration values obtained with the use of preamplification had lower discrepancy between the platforms (−0.01…0.37 vs. −0.22…0.6 log_10_ copies/µl).

Overall, the results of *H3F3A* K27M mutant and *H3F3A* wild-type target quantification obtained with the QIAcuity dPCR system were comparable with those obtained with the QX200 ddPCR platform, which indicates universality of the developed protocol.

## Discussion

Current standards for the diagnosis and management of CNS tumors include histological examination of tumor tissue followed by search for molecular markers [2]. Cell-free DNA in biological fluids represents an invaluable source of information on mutational landscape of a tumor. In neurooncology, alternative ways of studying genetic aberrations are crucially important, as conventional biopsies are unfeasible due to specific localization and nature of the tumor.

However, the use of liquid biopsies for CNS tumors is challenging. Firstly, there are no clear indications for molecular examination of CSF. The efficacy depends on combinations of many factors, including tumor characteristics (histopathology, grade, type of growth in relation to surrounding tissues and liquor spaces, metastatic potential), as well as the methods for CSF collection (ventricular access or lumbar puncture) and its timing (at manifestation, at recurrence, or during treatment). Secondly, in connection with the low stability of cells and cell-free nucleic acids in *ex vivo* CSF, concentration and quality of CSF-derived cfDNA should be expertly evaluated prior to genetic testing. It should be noted that representation of mutant alleles in cfDNA is normally reduced due to 1) intra-tumor heterogeneity and 2) the admixture of non-tumor cfDNA. According to Pan et al., concentrations of cfDNA obtained from CSF of patients with CNS tumors fall within a wide range of 5.5–1,845.0 pg/μl with a median of 25 pg/μl; notably, for high-grade tumors, diagnostic sensitivity to somatic variants in cfDNA is significantly higher [13]. Wang et al. have also demonstrated that the use of CSF-derived cfDNA for molecular profiling of low-grade CNS tumors is non-informative [19]. In our setting, concentrations of 150 bp fragments, indicating cfDNA suitable for dPCR analysis, were 0–15,170 copies/µl (median 35 copies/µl), with a tendency towards higher values observed for high-grade tumors.

As reported by Zhao et al., patients with glial tumors invading ventricular cavities or bordering subarachnoid cisterns have significantly higher CSF levels of cfDNA than patients with similar tumors confined within the anatomical boundaries of nervous tissue [20]. Connolly et al. have demonstrated unfeasibility of using CSF liquid biopsies for patients with intramedullary spinal ependymomas and associated it with negligible cfDNA diffusion into CSF, as the tumor is localized inside the spinal cord, beneath the pia mater which effectively separates the tumor mass from subarachnoid space [21]. In our setting, for pt. 7 with aggressive posterior fossa ependymoma group A, the ventricular CSF-derived cfDNA concentration was high (683 copies/µl) due to the vicinity of the fourth ventricle, despite low volume of the tumor and the absence of metastatic spread.

In the context of liquid biopsies, the topography of CSF sampling matters. Undoubtedly, the use of CSF samples obtained by relatively non-invasive methods from patients with suspected brain tumors would be a preferable option. Presence a ventriculoperitoneal shunt enables CSF collection with the minimal invasive burden. However, a closer look at published evidence on liquid biopsies from CNS tumors reveals the predominance of intraoperative sampling [13,19-21]. Mean cfDNA concentrations for intracranial CSF samples are significantly higher than for preoperative lumbar samples (e.g., 73.33 *cf.* 7.31 pg/μl, respectively [22]), hence the higher efficacy of genotyping for CSF samples collected intraoperatively*.*

Despite the significantly reduced concentrations of cfDNA in CSF derived by lumbar puncture, Fujioka et al. detected mutations in *IDH1*, *TERT*, or *H3* genes in six of seven high-grade glioma cases [22]*.* At the same time, On et al. were able to unequivocally identify *H3* K27M mutation in only 1 of 10 CSF samples obtained by lumbar puncture from newly diagnosed DMG patients; for three other cases in this cohort, the result was ambiguous, as only 1-2 mutant droplets were observed in ddPCR charts [23]. In our experience, concentrations of lumbar CSF-derived cfDNA (0–10, median 3 copies/µl) were also lower compared with the isolates obtained from ventricular CSF (0–15,170, median 94.5 copies/µl). This situation reflects lower levels of wild-type positivity observed in dPCR with lumbar CSF-derived templates. We encountered two lumbar CSF samples from *H3* K27M-positive DMG patients, similar to those described by On et al. [23]—with low cfDNA concentrations and single positive signals in dPCR charts. For one of them, ventricular CSF was also available, and the result was interpreted unambiguously (Figure 6). Indeed, in cases of extremely low cfDNA concentrations, interpretation of dPCR results may be challenging, as distinguishing between the non-specific trace degradation of the probe (sporadic high-fluorescence droplets) and weak specific signals is impossible.

In addition to the difficulties in CSF sample collection and primary handling, their advanced molecular analysis is sophisticated. Modern strategies for analysis of somatic mutations in cfDNA are based on the use of targeted high-throughput sequencing [12-14,19-21] or dPCR [14,16,21,23,25]. Apart from being an established method for detecting extremely low allelic loads, dPCR provides the unique ability to quantify target DNA without external standards. A number of technological platforms for dPCR are available, each of which has been comprehensively characterized on its own, whereas studies involving their comparison are scarce [24,25]. Here we demonstrate comparability for the highly competitive Droplet Digital™ PCR platform (Bio-Rad) and the relatively novel QIAcuity Digital PCR System (Qiagen). Quantitation of target at the start of dPCR involves a Poisson-based computational model. The value depends on three parameters: number of positive signals, total number of signals, and the reaction compartment volume. The two platforms used in this study operate under constant partition volume (droplet or plate cell). The total amount of signals (including both positive and negative) is defined by technical characteristics of the method—the counts of generated droplets for ddPCR™ or plate type (8.5k or 26k QIAcuity Nanoplate) for QIAcuity—and the number of replicates. The number of replicates, in turn, is limited by amount of cfDNA isolated from a CSF sample. The output of positive signals is determined solely by amount of target directly introduced in dPCR. Thus, increasing the absolute number of template molecules (as a sum total for all replicates) is the only means for enhancing the accuracy of analysis.

Preamplification step can be introduced in the protocol in order to increase representation of the target at the start of dPCR. This adjustment to the protocol is especially recommended for low quantities of starting material, as it ensures higher sensitivity of the analysis via target-specific enrichment. Increased numbers of target molecules at the start of the reaction allows it to outrun the background resulting from trace degradation of the probe [26]. The concentration measurements for the *H3* mutant and wild-type templates, obtained with alternative dPCR platforms by Bio-Rad and Qiagen, showed lower discrepancy upon the use of preamplification which, for its part, had no influence on the VAF estimates by dPCR.

Martínez-Ricarte et al. studied the mutational profile of CSF-derived cfDNA for a cohort of 20 patients with diffuse glioma. By using ddPCR, the authors identified *H3F3A* K27M in three patients with DMG, with VAFs within the range of 7.0–17.3% at cfDNA concentrations within the range of 4.8–37.5 pg/μl (with a median of 7.25 pg/μl) [14]. In a study by Stallard et al., a similar ddPCR-based approach afforded the detection of the *H3* K27M variant in liquid biopsies of high-grade glioma patients (*n* = 4) with significantly higher VAFs in cfDNA (18–65%) [16]. It should be noted that in the study by Martínez-Ricarte et al. CSF was collected by lumbar puncture, whereas in the study by Stallard et al. it was collected from liquor spaces of the brain. To emphasize the higher diagnostic value of ventricular CSF, Stallard et al. compared VAFs of *H3* K27M in lumbar and ventricular samples of CSF collected at autopsy from a patient with diffuse intrinsic pontine glioma. Indeed, concentration of target *H3* K27M variant in CSF collected from lateral ventricles was 2.5 times higher than in lumbar liquor (133 and 57 copies per ng cfDNA, respectively) [14,16].

## Conclusion

The study provides a reliable tissue-validated technical basis for detection of *H3* K27M somatic variant in CSF of pediatric patients with CNS tumors. The optimized universal dPCR protocol allows detection of solitary copies of the mutant allele with excellent analytical sensitivity. Thorough analytical characterization of cfDNA pools in terms of fragment lengths and concentrations is required to diminish the risk of false negatives. This requirement, along with insufficient quality and low representation of tumor DNA in CSF, limit standardization of the approach and its clinical prospects. Altogether, ventricular CSF sampling is more informative compared with lumbar sampling. Multicenter study designs are needed to evaluate clinical relevance of CSF liquid biopsies for rare CNS tumors.

## Data Availability

The original contributions presented in the study are included in the article/Supplementary Material, further inquiries can be directed to the corresponding author.

## Footnotes

1 The H3 K27M substitution is designated in accordance with the nomenclature of histone modification sites established in scientific literature. In the HGVS sequence variant nomenclature (http//www.hgvs.org), this variant corresponds to *H3F3A*: c.83A>T, p.(K28M).
